# Elevated mean arterial pressure and risk of impaired fasting glucose: a multicenter cohort study revealing age and sex interactions

**DOI:** 10.3389/fendo.2025.1580036

**Published:** 2025-06-18

**Authors:** Yuye Lin, Junzhong Zou, Miaoling Hong, Xudong Huang, Juan Wu

**Affiliations:** ^1^ Department of Clinical Laboratory, Jieyang People’s Hospital, Jieyang, Guangdong, China; ^2^ Precision Medicine Centre, Puning People’s Hospital, Puning, Guangdong, China

**Keywords:** mean arterial pressure, impaired fasting glucose, cohort study, age interaction, sex interaction

## Abstract

**Background:**

Evidence connecting mean arterial pressure (MAP) with impaired fasting glucose (IFG) was currently insufficient. The purpose of our research was to investigate how age and sex individually affect the relationship between MAP and the onset of IFG.

**Methods:**

Our study was a retrospective cohort analysis involving 184,291 participants from a multicenter health examination in China. The relationship between MAP and the onset of IFG was evaluated using Cox regression analysis. To further investigate the relationship, smooth curve fitting was applied to evaluate the dose-response association, while threshold effect analysis was performed to identify potential inflection points in MAP. Additionally, interaction effect analysis was conducted to examine whether age and sex modified the association between MAP and IFG risk.

**Results:**

The overall incidence of IFG was 11.28%. After multivariate adjustment, a significant positive association was observed between MAP and IFG risk (Hazard Ratio: 1.14, 95% Confidence Interval: 1.12–1.16, *P* < 0.001). Multivariate smooth splines analysis revealed a nonlinear relationship (*P* for nonlinearity < 0.05), with the curve slope decreasing as MAP exceeded 103.23 mmHg. Significant interaction effects between MAP and age or sex on IFG risk were also identified (*P* < 0.05).

**Conclusions:**

Our study revealed new insights into how MAP and IFG development were related, highlighting the influence of age and sex. These results stressed the need to consider MAP, age, and sex in IFG prevention, especially in high-MAP groups. Further investigation into the biological and behavioral mechanisms underlying these age- and sex-dependent relationships is warranted to inform personalized approaches for diabetes prevention.

## Background

Diabetes mellitus, a chronic metabolic disorder, has become a major public health challenge globally. It is estimated that around 422 million adults aged 30–79 years currently have diabetes, with the number continuing to increase (1). Projections suggest that the global population with diabetes will rise to 642 million by 2040 (2). Prediabetes, an intermediate state between normoglycemia and diabetes, includes impaired fasting glucose (IFG), impaired glucose tolerance, or a combination of both. It was showed that by 2021, approximately 726 million individuals worldwide were in a prediabetic state, with 298 million people suffering from IFG alone (3). IFG not only portends an increased risk of developing diabetes but is also closely associated with cardiovascular events such as atrial fibrillation and heart failure, as well as certain types of cancer (4, 5). Thus, restoring normal blood glucose levels in individuals with prediabetes is crucial. This intervention not only effectively delays or prevents the progression to diabetes but also significantly reduces all-cause mortality, ultimately enhancing the long-term prognosis and quality of life for those affected (2).

While current therapeutic paradigms predominantly target glycemic control, novel research reveals that hemodynamic disturbances, especially blood pressure dysregulation, may constitute an underrecognized pathogenic component in IFG progression (6). Hypertension, defined by elevated systemic arterial pressure, is typically characterized using systolic blood pressure (SBP) and diastolic blood pressure (DBP) (7). However, accumulating evidence suggests that the predictive capacities of SBP and DBP may differ across various age groups (8, 9). Mean arterial pressure (MAP) is a direct and effective method for assessing cardiovascular function, as it represents the average arterial blood pressure over a cardiac cycle. Recent studies have demonstrated that MAP exhibits superior performance in identifying hypertension, with an accuracy rate as high as 95.2%, compared to SBP and DBP alone (10). While elevated SBP and DBP are established risk factors for diabetes, MAP, as an integrative measure of both SBP and DBP, has been proposed to be equally potent in predicting diabetes progression (11, 12). This notion is further supported by findings from the Chinese population, where MAP has been shown to be significantly associated with the risk of incident diabetes (13, 14). Moreover, research has highlighted that cumulative exposure to elevated SBP and DBP is correlated with impaired glucose tolerance (15). Extending these observations, we hypothesize that MAP may serve as a significant indicator for IFG, thereby warranting further investigation into its potential role in diabetes risk stratification. Numerous studies have identified sex and age as significant factors influencing the incidence of non-communicable diseases (16). The role of sex in diabetes incidence remains a topic of debate (17). Among White, South Asian, Black, and Chinese populations, men generally exhibit higher risks of hypertension and diabetes compared to women (18–20). However, in the United States, no significant sex differences in diabetes prevalence have been observed (21). Conversely, a study focusing on Hispanic and Latino children and adolescents revealed that two-thirds of individuals diagnosed with diabetes were female (22). Given these findings, investigating the associations between MAP and IFG across different sexes and ages is of substantial significance.

Our study provided new evidence on the relationship between MAP and IFG risk, a finding previously unreported in large-scale multicenter cohort studies. Furthermore, we clarified how age and sex modify this relationship. These findings underscore the importance of tailored prevention strategies, particularly for high-MAP individuals, filling a key knowledge gap in diabetes prevention. Implementing IFG risk monitoring based on MAP, tailored to sex and appropriate screening timing, can facilitate early identification and targeted interventions for diabetes prevention, ultimately reducing the prevalence of prediabetes and diabetes.

## Method

### Study design and population

We employed the multicenter health check-up data from the Rich Healthcare Group, as supplied by Chen et al. (23), following the DRYAD database's terms of use and the Creative Commons Attribution-NonCommercial-Share-Alike 4.0 International license. The original study aimed to explore the association between body mass index (BMI) and age with incident diabetes among adults. It included 211,833 participants who underwent at least two health check-ups between 2010 and 2016. Participants were excluded if they had incomplete data regarding sex, age, or fasting plasma glucose (FPG) (n=135,317). Those with a BMI exceeding 55 kg/m² or below 15 kg/m² were also excluded (n=152). Furthermore, individuals with a follow-up period shorter than 2 years (n=324,233) were excluded. Additionally, individuals already diagnosed with diabetes (n=7,112) or with an indeterminate diabetes status (n=6,630) were excluded.

**Figure 1:**
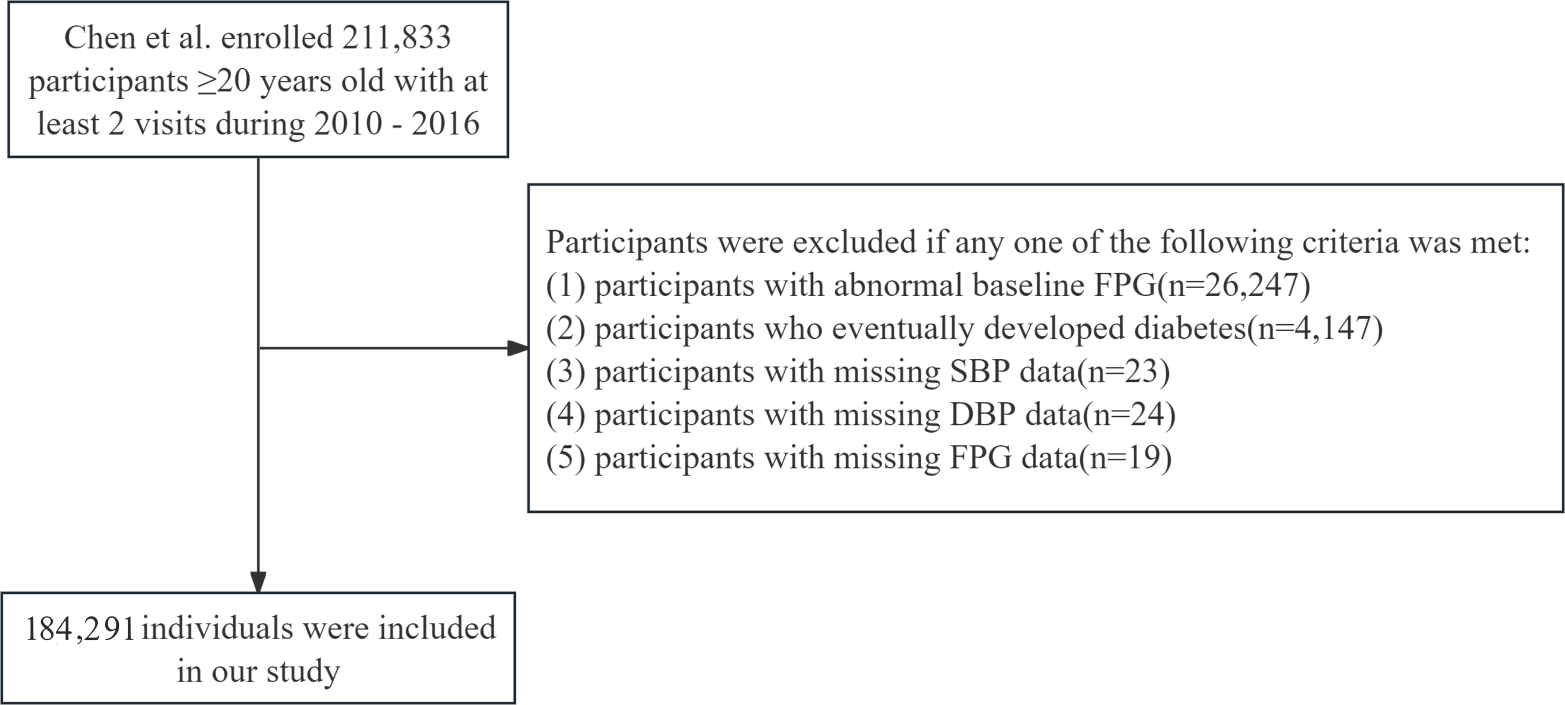
Flowchart of population inclusion and exclusion. SBP, systolic blood pressure; DBP, diastolic blood pressure; FPG, fasting plasma glucose.

For our secondary analysis, we focused on participants with normal baseline FPG levels to investigate the impact of baseline MAP on the incidence of IFG. We excluded participants with abnormal baseline FPG levels (n=26,247), participants who eventually developed diabetes (n=4,147), missing SBP data (n=23), missing DBP data (n=24), and missing FPG data during follow-up (n=19). The participant selection process was illustrated in **Figure 1**.

### Definition

In our study, MAP served as the exposure variable, calculated using the formula: MAP = (1/3) SBP + (2/3) DBP. Blood pressure measurements were obtained by trained staff using a standard mercury sphygmomanometer.

The primary outcome of interest was IFG, defined according to the American.

Diabetes Association criteria as a FPG level ranging from 5.6 to 7.0 mmol/L, in the absence of a diabetes diagnosis.

Hyperlipidemia was defined by the presence of any of the following lipid profiles: triglycerides (TG) ≥ 2.3 mmol/L, total cholesterol (TC) ≥ 6.2 mmol/L, high-density lipoprotein cholesterol (HDL-C) ≤ 1.0 mmol/L, or low-density lipoprotein cholesterol (LDL-C) ≥ 4.1 mmol/L (24). Normal blood pressure was defined as SBP< 120 mmHg and DBP< 80 mmHg (25).

Follow-up duration was calculated from the date of the participants' first health check-up to the date of their last check-up.

### Covariates

The potential confounders adjusted for in our study included demographic factors (sex, age), anthropometric measures (BMI), biochemical parameters (FPG, TC, TG, LDL, HDL, alanine aminotransferase [ALT], aspartate aminotransferase [AST], blood urea nitrogen [BUN], creatinine [CCR]), lifestyle factors (smoking and alcohol consumption status), and family history of diabetes.

Participants' demographic information, lifestyle habits, and family history of diseases were collected via questionnaires. Physical measurements, including height, weight, and blood pressure, were taken by trained staff. BMI was calculated as weight in kilograms divided by height in meters squared. Smoking and drinking status were analyzed as categorical variables (current, former, never) as recorded in the original dataset. Prior to fasting blood draws, participants were required to fast for at least 10 hours. Biochemical parameters were measured using a Beckman 5800 automated biochemical analyzer.

### Statistical handling of missing data

As illustrated in **Figure 1**, a total of 184,291 participants were ultimately included in our study. Missing data distributions among principal covariates were quantified as follows: ALT (83.62%, n=1,541); AST (58.45%, n=107,709); BUN (10.08%, n=18,570); CCR (5.30%, n=9,766); TC (2.29%, n=4,231); HDL (45.26%, n=83,401); LDL (44.98%, n=82,898); TG (2.30%, n=4,244); with lifestyle covariates showing substantial missingness - smoking status and alcohol consumption (both 72.32%, n=133,279 each).

To address missing data limitations effectively while preserving statistical power and reducing potential selection bias, we applied multiple imputation techniques to all continuous variables exhibiting less than 50% missing observations. Implementation followed established methodological frameworks described by Van Buuren and Groothuis-Oudshorn (26), utilizing the R mice package with five imputed datasets generated through chained equations incorporating predictive mean matching (26). Continuous variables were imputed using distribution-appropriate measures, with means employed for normally distributed data and medians for skewed distributions. For AST, which exhibited >50% missingness, we transformed this continuous variable into a categorical one through tertile categorization. Regarding categorical variables with missingness exceeding 50%, we preserved the missing data structure by creating a distinct 'missing' category. This comprehensive approach enabled maximal utilization of available data while maintaining analytical validity and minimizing information loss.

### Statistical method

The normality of variables was assessed using a combination of histogram distributions, prior literature, and the distribution of means and standard deviations (SD). Continuous variables that followed a normal distribution were described using mean ± SD, while those with a skewed distribution were reported as median (interquartile range). Categorical variables were presented as frequencies (%). For comparing differences among groups, the chi-square test was applied to categorical variables, One-Way ANOVA was used for normally distributed continuous variables, and the Kruskal-Wallis H test was employed for continuous variables with skewed distributions.

Hazard ratios (HRs) and 95% confidence intervals (CIs) for IFG incidence were estimated using multivariate Cox regression analysis. The proportional hazards assumption was verified using the Schoenfeld residuals test, with results confirming its validity (*P* > 0.05). Kaplan–Meier survival curves were used to evaluate the IFG outcome based on the quantiles of MAP, with comparisons made using the log-rank test. MAP was incorporated into the Cox regression analysis both as a categorical variable (divided into four quantiles) and as a continuous variable (per 10-unit increase). Three models were constructed for the analysis: Model 1 unadjusted for any confounders. Model 2 adjusted for age, sex, BMI, and FPG. Building upon Model 2, Model 3 additionally adjusted for TC, TG, HDL, LDL, AST, ALT, BUN, CCR, smoking status, drinking status, and family history of diabetes. MAP was categorized into quartiles, and the *P* for trend was calculated to validate the results obtained with MAP as a continuous variable and to explore potential nonlinearity. Sensitivity analyses were performed by restricting the analysis to participants who were nonsmokers or nondrinkers. Additionally, we conducted a multivariate regression analysis using the original dataset with missing values to verify the robustness of the multiple imputation results. Effect sizes and *P*-values derived from these models were calculated and compared.

To further verify the stability of the outcomes, subgroup analyses were performed according to sex, age, BMI, lipid level and family history of diabetes. The potential impact of unmeasured confounding factors was evaluated by calculating the E-value to assess the robustness of the findings.

To examine the potential nonlinear relationship between MAP and IFG, we applied a restricted cubic spline model to generate smooth curves. MAP was modeled as a continuous variable with four knots positioned at the 5th, 35th, 65th, and 95th percentiles, in accordance with Harrell's guidelines (27). The likelihood ratio test was used to evaluate nonlinearity by comparing models with and without cubic spline terms. When non-linear associations were detected, a two-piecewise regression model was employed to identify the threshold effect of MAP on IFG.

Upon establishing the independent association between MAP and IFG, we proceeded to investigate potential interaction effects of MAP and IFG across different sex and age subgroups. We evaluated multiplicative interactions (MAP×age; MAP×sex) by comparing models incorporating cross-product interaction terms of age or sex with MAP levels, utilizing likelihood ratio tests to assess the significance of these interaction terms for each outcome. Furthermore, we examined additive interactions to assess the combined impact of MAP and age or sex on the risk of IFG occurrence. For the analysis of additive interactions, we computed the relative excess risk due to interaction (RERI) and the attributable proportion (AP). RERI indicates whether the combined effect of MAP and age or sex surpasses the sum of their individual effects, while AP estimates the proportion of excess risk attributable to the joint exposure of MAP and age or sex.

### Statistical software

Statistical analyses were conducted using R Statistical Software (Version 4.2.2, http://www.R-project.org, The R Foundation) and the Free Statistics analysis platform (Version 2.0, Beijing, China, http://www.clinicalscientists.cn/freestatistics). Statistical significance was determined by a two-sided *P* value less than 0.05.

## Result

### Baseline characteristics

Our study encompassed 184,291 eligible participants whose ages ranged from 20 to 97 years. The overall prevalence of IFG in this cohort was 11.28%. **Table 1** delineated the general characteristics of the participants, categorized based on whether their blood pressure was normal or abnormal. Compared with participants with normal blood pressure, those with abnormal blood pressure were found to be older, predominantly male, and had lower HDL-C levels. Additionally, they exhibited higher values for BMI, FPG, TC, TG, LDL, HDL, ALT, AST, BUN, and CCR (all *P* < 0.05).

**Table 1:** Baseline demographic and clinical characteristics of subjects stratified by blood pressure.

Characteristics	Total	Blood pressure		*P* value
		Normal	Abnormal	
Number	184,291	97,032	87,259	
Age (Years)	41.0 ± 12.1	38.6 ± 9.8	43.7 ± 13.7	< 0.001
Sex				< 0.001
Male	97,809 (53.1)	39,947 (41.2)	57,862 (66.3)	
Female	86,482 (46.9)	57,085 (58.8)	29,397 (33.7)	
BMI (kg/m^2^)	23.0 ± 3.3	22.1 ± 2.9	24.0 ± 3.3	< 0.001
SBP (mm Hg)	117.8 ± 15.8	106.4 ± 8.1	130.6 ± 12.1	< 0.001
DBP (mm Hg)	73.5 ± 10.6	67.0 ± 6.5	80.8 ± 9.5	< 0.001
FPG (mmol/L)	4.8 ± 0.5	4.7 ± 0.5	4.8 ± 0.5	< 0.001
TC (mmol/L)	4.7 ± 0.9	4.6 ± 0.8	4.8 ± 0.9	< 0.001
TG (mmol/L)	1.3 ± 0.9	1.1 ± 0.7	1.5 ± 1.1	< 0.001
HDL-C (mmol/L)	1.4 ± 0.3	1.4 ± 0.3	1.3 ± 0.3	< 0.001
LDL-C (mmol/L)	2.7 ± 0.7	2.7 ± 0.6	2.8 ± 0.7	< 0.001
ALT (U/L)	23.2 ± 21.7	20.1 ± 20.2	26.7 ± 22.9	< 0.001
BUN (mmol/L)	4.6 ± 1.2	4.5 ± 1.1	4.7 ± 1.2	< 0.001
CCr (μmol/L)	69.7 ± 15.7	66.9 ± 14.8	72.8 ± 16.2	< 0.001
Family history of diabetes				< 0.001
No	180,644 (98.0)	94,868 (97.8)	85,776 (98.3)	
Yes	3,647 (2.0)	2,164 (2.2)	1,483 (1.7)	
Smoking status				< 0.001
Current smoker	9,672 (5.2)	4,115 (4.2)	5,557 (6.4)	
Ever smoker	2,120 (1.2)	870 (0.9)	1,250 (1.4)	
Never smoker	39,220 (21.3)	20,601 (21.2)	18,619 (21.3)	
Not recorded	133,279 (72.3)	71,446 (73.6)	61,833 (70.9)	
Drinking status				< 0.001
Current drinker	988 (0.5)	344 (0.4)	644 (0.7)	
Ever drinker	7,370 (4.0)	3,198 (3.3)	4,172 (4.8)	
Never drinker	42,654 (23.1)	22,044 (22.7)	20,610 (23.6)	
Not recorded	133,279 (72.3)	71,446 (73.6)	61,833 (70.9)	
AST (Tertiles)				< 0.001
Tertile 1	25,378 (13.8)	15,969 (16.5)	9,409 (10.8)	
Tertile 2	25,487 (13.8)	13,374 (13.8)	12,113 (13.9)	
Tertile 3	25,717 (14.0)	10,616 (10.9)	15,101 (17.3)	
Not recorded	107,709 (58.4)	57,073 (58.8)	50,636 (58)	
MAP (mmHg)	88.3 ± 11.4	80.1 ± 6.2	97.4 ± 8.7	< 0.001

Mean ± standard deviations or median (quartile 1, quartile 3) was used to summarize continuous variables, while categorical variables were expressed as n (%).

BMI, body mass index; SBP, systolic blood pressure; DBP, diastolic blood pressure; FPG, fasting plasma glucose; TC, total cholesterol; HDL-C, high-density lipoprotein cholesterol; LDL-C, low-density lipoprotein cholesterol; ALT, alanine aminotransferase; AST, aspartate aminotransferase; BUN, blood urea nitrogen; CCr, creatinine; TG, triglyceride; MAP, mean arterial pressure.

### Association between the MAP and the IFG events

**Figure 2:**
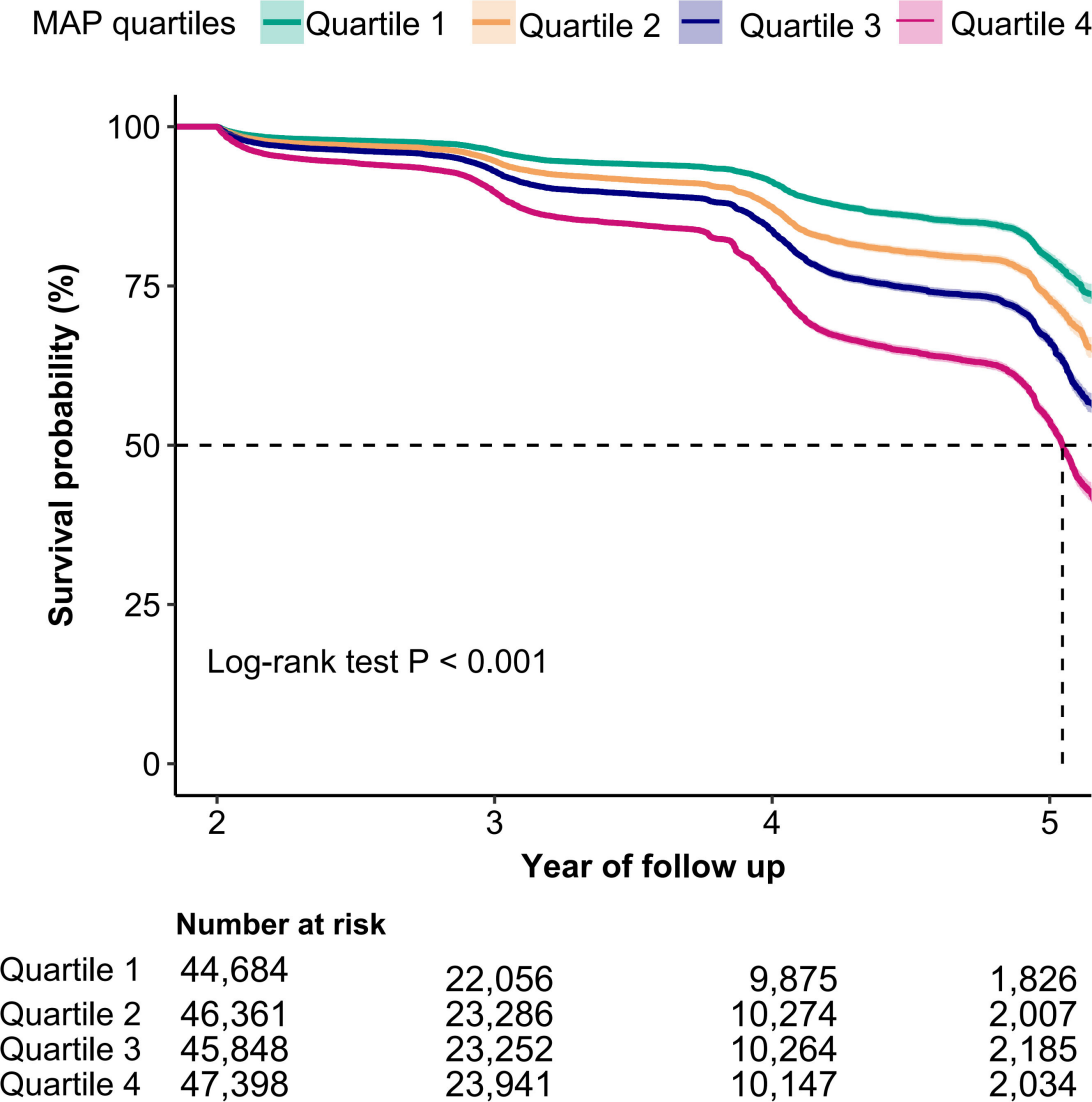
Kaplan-Meier curves for the probability of IFG base on MAP quartiles. MAP, mean arterial pressure; IFG, impaired fasting glucose. A log-rank test *P* value < 0.001 indicated a statistically significant difference in incident IFG across MAP quartile groups.

For the IFG individuals who were entered into follow-up observation, the cumulative hazard of IFG was higher in MAP quartile 4 compared to the other three groups, as illustrated by the Kaplan-Meier curves (Log-rank test *P*<0.001, **Figure 2**). The multivariate Cox proportional hazards model, adjusted for potential confounders, demonstrated that a 10-unit increment in MAP raised the incidence of IFG by 14% (HR: 1.14, 95% CI: 1.12–1.16, *P*<0.001) (**Table 2**, Model 3). In the multivariable Cox proportional hazards models, the risk of IFG occurring in quartile 4 was 1.54 times that of the quartile 1 group, after adjustment for potential confounders (HR: 1.54, 95% CI: 1.45–1.64, *P*<0.001) (**Table 2**, Model 3).

**Table 2:** The association between MAP and the risk of IFG in different models.

MAP (mmHg)	Numbers	Event (%)	Model 1		Model 2		Model 3	
			HR (95% CI)	*P* value	HR (95% CI)	*P* value	HR (95% CI)	*P* value
Per 10-mmHg increase	184,291	20,783 (11.3)	1.38 (1.37–1.40)	<0.001	1.14 (1.13–1.16)	<0.001	1.14 (1.12–1.16)	<0.001
MAP quartiles								
Quartile 1	44,684	2,740 (6.1)	1.00 (Ref)		1.00 (Ref)		1.00 (Ref)	
Quartile 2	46,361	4,127 (8.9)	1.43 (1.36–1.50)	<0.001	1.17 (1.11–1.23)	<0.001	1.18 (1.11–1.26)	<0.001
Quartile 3	45,848	5,467 (11.9)	1.88 (1.79–1.97)	<0.001	1.28 (1.23–1.35)	<0.001	1.31 (1.23–1.40)	<0.001
Quartile 4	47,398	8,449 (17.8)	2.87 (2.75–2.99)	<0.001	1.52 (1.45–1.59)	<0.001	1.54 (1.45–1.64)	<0.001
*P* for trend				<0.001		<0.001		<0.001

BMI, body mass index; SBP, systolic blood pressure; DBP, diastolic blood pressure; FPG, fasting plasma glucose; TC, total cholesterol; HDL-C, high-density lipoprotein cholesterol; LDL-C, low-density lipoprotein cholesterol; ALT, alanine aminotransferase; AST, aspartate aminotransferase; BUN, blood urea nitrogen; CCr, creatinine; TG, triglyceride; MAP, mean arterial pressure; IFG, impaired fasting glucose; 95% CI, 95% confidence interval; HR, hazard ratio; Ref, reference;

Model 1: Not adjusted for any confounders.

Model 2: Adjusted for age, sex, BMI, FPG.

Model 3: Adjusted for Model 2+TC, TG, LDL, HDL, ALT, AST, BUN, CCR, family history of diabetes, smoking status, drinking status.

Upon excluding participants with a history of smoking or alcohol consumption, the association between MAP and IFG remained positively associated. Sensitivity analyses, which excluded participants with missing data, continued to reveal a significant positive association between MAP and IFG (HR: 1.15, 95% CI: 1.13–1.18) (see **Supplementary Table S1**).

**Figure 3:**
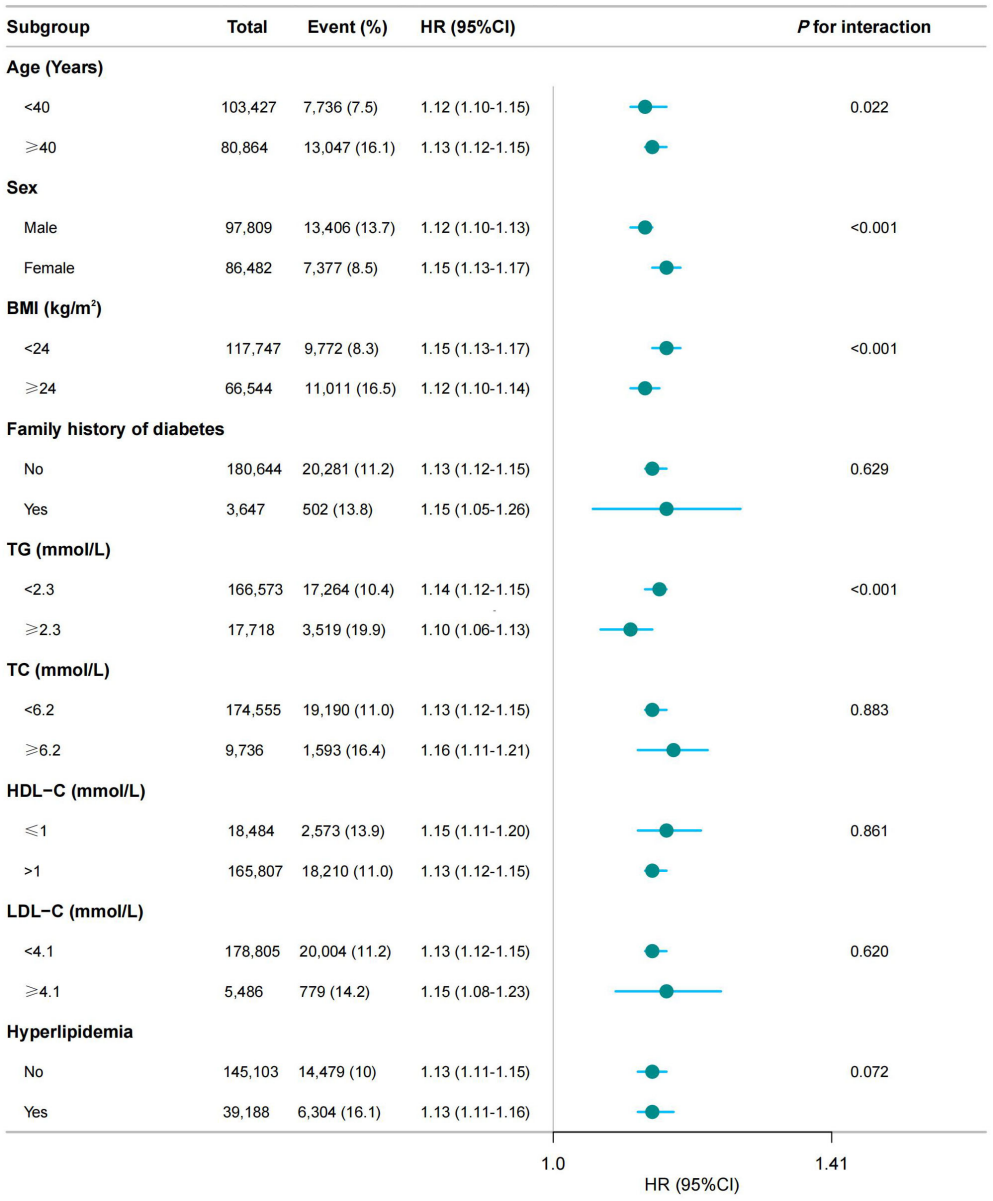
Subgroup analysis of the association between MAP and the risk of IFG. BMI, body mass index; SBP, systolic blood pressure; DBP, diastolic blood pressure; FPG, fasting plasma glucose; TC, total cholesterol; HDL-C, high-density lipoprotein cholesterol; LDL-C, low-density lipoprotein cholesterol; ALT, alanine aminotransferase; AST, aspartate aminotransferase; BUN, blood urea nitrogen; CCr, creatinine; TG, triglyceride; MAP, mean arterial pressure; IFG, impaired fasting glucose. The model adjusted for age, sex, BMI, FPG, TC, TG, LDL, HDL, ALT, AST, BUN, CCR, smoking status, drinking status, and family history of diabetes (except when family history of diabetes was the stratification variable). A significant interaction (*P* < 0.05) indicated heterogeneity in the association between MAP and IFG risk across subgroups.

In addition to the primary analysis, we conducted stratified analyses based on age, sex, BMI, lipid level, and family history of diabetes. As expected, we observed a statistically significant association between MAP and IFG across all subgroups. However, for incident IFG, we detected significant variations in age, sex, TG and BMI (*P* < 0.05 for interaction) within the subgroups (see **Figure 3**).

While examining the relationship between MAP and IFG events, we assessed the potential impact of unmeasured confounding factors by calculating the E-value. The study results indicated that if there was an unmeasured confounding factor, its relative risk association with both IFG events and MAP would need to reach 1.54 to fully explain the observed association between MAP and IFG events.

### Curve fitting and inflection point analysis

**Figure 4:**
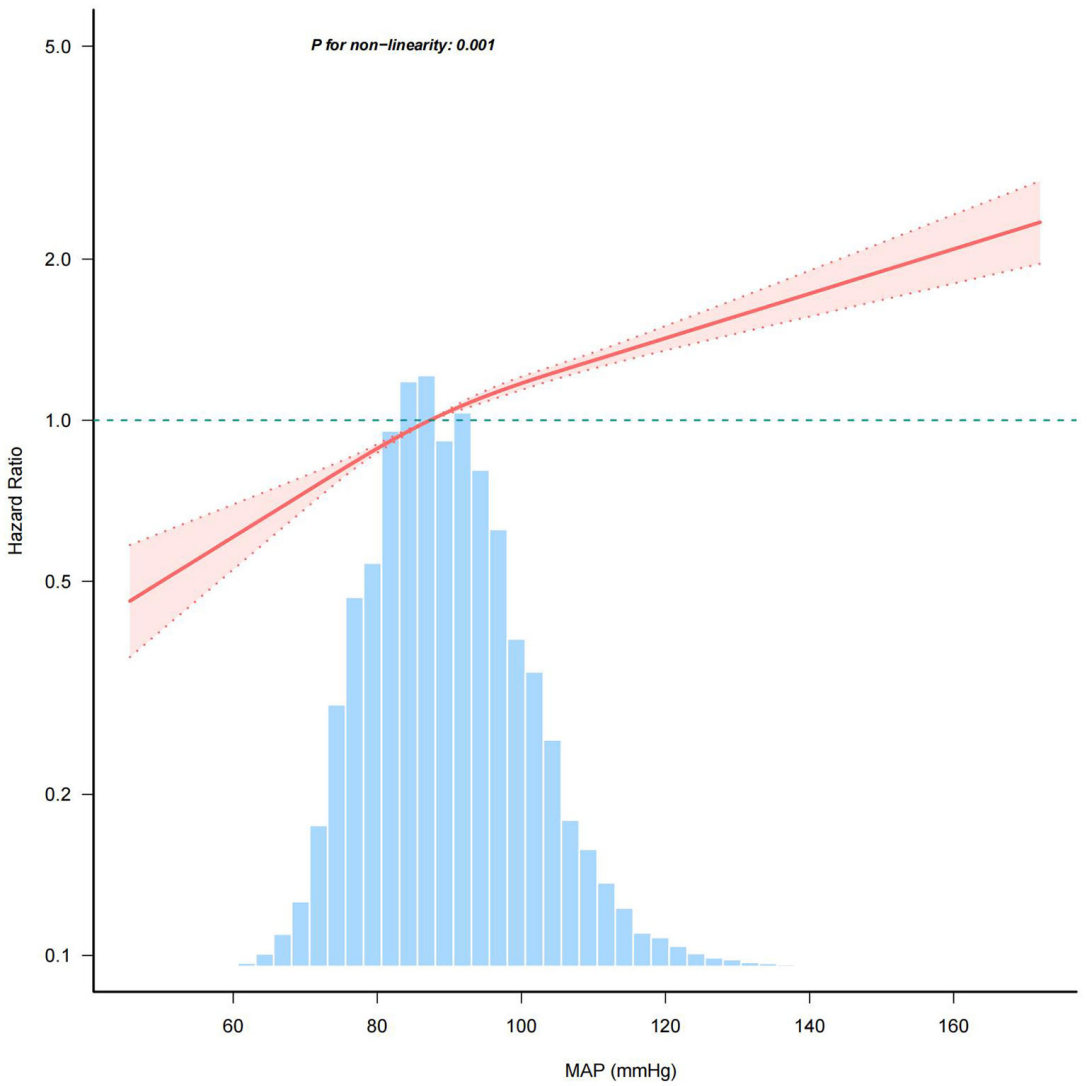
Association between MAP and the risk of IFG. BMI, body mass index; SBP, systolic blood pressure; DBP, diastolic blood pressure; FPG, fasting plasma glucose; TC, total cholesterol; HDL-C, high-density lipoprotein cholesterol; LDL-C, low-density lipoprotein cholesterol; ALT, alanine aminotransferase; AST, aspartate aminotransferase; BUN, blood urea nitrogen; CCr, creatinine; TG, triglyceride; MAP, mean arterial pressure; IFG, impaired fasting glucose. The model adjusted for age, sex, BMI, FPG, TC, TG, LDL, HDL, ALT, AST, BUN, CCR, smoking status and family history of diabetes. The percentage density distribution of MAP across the study's participants is shown by the light blue histograms. A reference HR value of 1.0 is indicated by the horizontal dotted lines, which serve as a benchmark. The bold central lines represent the estimated adjusted hazard ratios, and the shaded areas around them denote the 95% CIs. A *P*-value for non-linearity < 0.05 indicated a non-linear association between MAP and IFG risk.

Multivariable-adjusted restricted cubic spline analyses revealed a distinct positive relationship between MAP and the prevalence of IFG, with statistically significant nonlinearity (*P* for nonlinearity < 0.05, **Figure 4**). MAP was modeled as a continuous variable, with adjustments for covariates in Model 3. The prevalence of IFG increased with rising MAP levels until reaching a critical point at 103.23. Below this MAP value, the prevalence of IFG rose with increasing MAP (HR: 1.014, 95% CI: 1.012–1.016). Conversely, beyond the inflection point of 103.23, the dose-response curve indicated a plateau (HR: 1.008, 95% CI: 1.004–1.013) (see **Table 3**).

**Table 3:** Threshold effect of MAP on the incidence of IFG.

MAP (mmHg)	HR (95%CI)	*P* value
<103.23	1.014 (1.012–1.016)	< 0.001
≥103.23	1.008 (1.004–1.013)	< 0.001
Likelihood Ratio test		<0.001

BMI, body mass index; SBP, systolic blood pressure; DBP, diastolic blood pressure; FPG, fasting plasma glucose; TC, total cholesterol; HDL-C, high-density lipoprotein cholesterol; LDL-C, low-density lipoprotein cholesterol; ALT, alanine aminotransferase; AST, aspartate aminotransferase; BUN, blood urea nitrogen; CCr, creatinine; TG, triglyceride; MAP, mean arterial pressure; IFG, impaired fasting glucose; 95% CI, 95% confidence interval; HR, hazard ratio.

The model adjusted for age, sex, BMI, FPG, TC, TG, LDL, HDL, ALT, AST, BUN, CCR, smoking status and family history of diabetes. A significant likelihood ratio test (*P* < 0.05) supported the existence of a threshold effect, indicating that the association between MAP and the incidence of IFG was nonlinear with an inflection point at 103.23.

### Age and sex affect the association between MAP and IFG status

**Table 4** showed that there was a significant interaction between sex and age with MAP and IFG status, with both multiplicative and additive interactions achieving statistical significance (*P* < 0.001). Participants aged≥40 years exhibited substantially higher HRs than younger counterparts, with high MAP conferring an HR of 1.98 (95% CI: 1.90–2.07) versus 1.25 (95% CI: 1.19–1.31) in the <40 group when compared to respective low-MAP references. Interaction analysis indicated that the combined effect of age and MAP was markedly greater than the simple sum of their individual effects. Specifically, when age and MAP jointly influence IFG events, the RERI was 30% (HR: 0.30, 95% CI: 0.22–0.37), of which 15% of the risk can be attributed to their interaction (HR: 0.15, 95% CI: 0.11–0.19).

**Table 4:** Multiplicative and additive interactions between MAP and different sex and age groups on the risk of IFG.

Variable	MAP status	HR (95% CI)	*P* for interaction	Additive interaction		Multiplicative interaction
				RERI (95% CI)	AP (95% CI)	HR (95% CI)
Age			<0.001	0.30 (0.22–0.37)	0.15 (0.11–0.19)	1.11 (1.04–1.17)
<40	Low	1.00 (Ref)				
	High	1.25 (1.19–1.31)				
≥40	Low	1.43 (1.36–1.50)				
	High	1.98 (1.90–2.07)				
Sex			<0.001	0.20 (0.15–0.26)	0.18 (0.13–0.23)	1.27 (1.20–1.35)
Female	Low	1.00 (Ref)				
	High	1.15 (1.11–1.20)				
Male	Low	0.76 (0.73–0.81)				
	High	1.12 (1.07–1.18)				

BMI, body mass index; SBP, systolic blood pressure; DBP, diastolic blood pressure; FPG, fasting plasma glucose; TC, total cholesterol; HDL-C, high-density lipoprotein cholesterol; LDL-C, low-density lipoprotein cholesterol; ALT, alanine aminotransferase; AST, aspartate aminotransferase; BUN, blood urea nitrogen; CCr, creatinine; TG, triglyceride; MAP, mean arterial pressure; IFG, impaired fasting glucose; 95% CI, 95% confidence interval; HR, hazard ratio; RERI, relative excess risk due to interaction; AP, attributable proportion.

The analysis adjusted for age (not for the age groups), sex, BMI, FPG, TC, TG, LDL, HDL, ALT, AST, BUN, CCR, family history of diabetes, smoking status, drinking status.

Elevated MAP was significantly associated with increased IFG risk in both sexes, although baseline susceptibility varied considerably between different sexes, with males serving as the reference group and females exhibiting a lower baseline risk (HR: 0.76) in low-MAP groups. Stratified analyses revealed sexually dimorphic response patterns: males experienced a 15% higher risk of IFG at elevated MAP levels (HR: 1.15, 95% CI: 1.11–1.20) compared to their low-MAP counterparts. In contrast, females, despite their lower baseline risk (HR: 0.76, 95% CI: 0.73–0.81 for low MAP), demonstrated a 47% relative increase in IFG risk when subjected to high MAP (HR: 1.12, 95% CI: 1.07–1.18). The presence of both female sex and high MAP resulted in a 20% higher risk of IFG than the sum of their independent effects (RERI: 0.20, 95% CI: 0.15–0.26), with 18% of the risk attributable to the interaction between sex and MAP (AP: 0.18, 95% CI: 0.13–0.23). This paradoxical pattern indicated that, although men have a natural predisposition to IFG, their metabolic response to elevated MAP results in a disproportionate change in risk. Conversely, females exhibited greater hemodynamic sensitivity, whereby increases in blood pressure conferred an amplified relative risk compared to their baseline profiles.

## Discussion

To the best of our knowledge, this large retrospective cohort study of a Chinese population undergoing physical examinations is the first to consistently observe a significant positive association between MAP and the risk of IFG. Subgroup analysis and sensitivity analysis showed stable results. Notably, our findings suggested a dose-response relationship, indicating that MAP was linked to IFG in a non-linear manner (*P* for nonlinearity < 0.05), with the relationship being more pronounced at MAP levels around 103.23 mmHg. Moreover, our study revealed significant interaction effects of MAP with both sex and age on IFG risk, demonstrating that these demographic factors critically modify the association between blood pressure dynamics and glucose metabolism. Specifically, both males and females with high MAP faced an increased risk, although the effect was slightly more pronounced in females. The combination of older age and high MAP was particularly concerning. This finding underscores the importance of personalized risk assessment, as the impact of elevated MAP on IFG development varies meaningfully across different age groups and between sexes. These results underscored the complexity of the relationship between these factors in determining health outcomes.

Epidemiological research has firmly established MAP as an independent risk factor for diabetes. For instance, a cohort study of a Chinese population undergoing physical examinations has confirmed the positive association between MAP and diabetes (14). Additionally, a recent cohort study in rural China further explored the sex differences in the association between MAP and diabetes among 12,284 study participants. They found a significant association between MAP and diabetes in women under 60 years of age, while this conclusion did not hold for men and women aged 60 and older (13). Currently, there is still a lack of evidence regarding the association between MAP and prediabetes. Given the large number of individuals with prediabetes and its potential impact on public health, investigating the influencing factors for the occurrence of prediabetes holds significant clinical value. In our research, which involved 184,291 participants undergoing health examinations in China, we have established a significant association between MAP and IFG. Furthermore, our study aimed to investigate how age and sex might modify the effect of MAP on the risk of developing IFG.

Our study has revealed several key insights. Firstly, our research suggested a positive association between MAP and IFG. Further research is essential to validate our findings and explore the intricate relationship and potential underlying mechanisms. Notably, when MAP exceeds 103.23 mmHg, the strength of the association between MAP and the risk of IFG diminishes. The specific mechanism behind this phenomenon is currently unclear, but it may be related to the fact that individuals with higher MAP are more likely to have additional complications, such as metabolic abnormalities and fatty liver disease. These conditions may have a more significant impact on glucose metabolism than MAP itself (28, 29). Therefore, we think that individuals with MAP <103.23 mmHg should monitor glycemic changes vigilantly, as this pressure range demonstrates a stronger association with IFG risk, even in the absence of hypertension. For those exceeding this threshold, while the association with IFG risk may attenuate, rigorous blood pressure management remains imperative due to persistent cardiovascular risks. Notably, the magnitudes of the significant associations between MAP and incident IFG were modest but consistent across different event subtypes, including subgroups stratified by age, sex, BMI, lipid level, and family history of diabetes. Upon further analysis, we discovered that the measures of interaction provide a more nuanced perspective on how MAP interacts with age and sex. For age, both additive and multiplicative interactions were positively associated. The influence of elevated MAP was more significant in individuals aged over 40 years. Consequently, it is recommended that individuals in this age group, particularly those diagnosed with hypertension (elevated MAP), be prioritized for monitoring. For these individuals, more stringent blood pressure management targets than the standard threshold should be considered, and lifestyle interventions should be initiated proactively. With respect to sex differences, although the individual impact of high MAP was more pronounced in males, the combined effect of male sex and elevated MAP resulted in a risk increase that was lower than anticipated. This finding suggests that, despite men having a higher susceptibility to IFG, the incremental contribution of elevated MAP to their risk is relatively diminished, exerting a more substantial effect on women's IFG risk. Therefore, when assessing the impact of elevated MAP on males, it is crucial to consider the moderating effect of sex interaction to accurately evaluate the actual risk. Clinically, it may be necessary for women to commence blood pressure interventions earlier to mitigate the risk of developing pre-diabetes. More studies are needed to provide deeper insights into the interplay between these factors and their association with prediabetes susceptibility.

Additionally, various other pathogenic mechanisms related to MAP may help us understand the role of MAP in glucose metabolism. People with prediabetes often present with hypertension or high MAP, which may be associated with common pathophysiological mechanisms of insulin resistance and metabolic syndrome. Dysfunction of pancreatic β-cells is a key factor in the onset and progression of diabetes (30). Elevated MAP may increase peripheral vascular resistance and reduce blood flow perfusion to peripheral tissues, thereby affecting glucose breakdown and utilization, which in turn increases the risk of diabetes. Additionally, elevated blood pressure can further exacerbate insulin resistance by impacting endothelial function (31). It is noteworthy that hypertension is closely associated with elevated levels of inflammatory markers. These inflammatory factors may exacerbate insulin resistance by interfering with insulin signaling pathways (32). Long-term insulin resistance places an increased burden on pancreatic β-cells, ultimately leading to β-cell dysfunction and failure, which contributes to abnormal glucose metabolism and elevated blood sugar levels. Additionally, with advancing age, the risk of insulin resistance and β-cell dysfunction increases, while the likelihood of insufficient sleep and poor nutrition also rises significantly, further elevating the risk of abnormal glucose metabolism (33). This indicates that the timing of screening and timely intervention are crucial for the prevention of diabetes. According to published data, there were notable differences in the incidence of diabetes between sexes, with men generally experiencing a higher prevalence than women, particularly among middle-aged populations (21, 34). Consistent with previous studies, the prevalence of IFG in men was 13.7%, which is higher than the 8.5% prevalence observed in women. The mechanisms underlying this difference may be related to variations in insulin resistance between sexes. In women, abnormalities in glucose metabolism are primarily associated with peripheral insulin resistance, leading to diabetes characterized by impaired glucose tolerance. In contrast, the etiology of diabetes in men is mainly driven by hepatic insulin resistance, which primarily results in impaired fasting blood glucose levels. These sex differences highlight the importance of considering sex-specific factors in the prevention and treatment of diabetes (35, 36). One plausible explanation for this observation is the influence of sex hormones and epigenetic factors. In women, steroid hormones play a beneficial role in maintaining glucose homeostasis. Elevated plasma testosterone levels are also considered a risk factor for diabetes in women. Conversely, low plasma testosterone levels are recognized as a risk factor for diabetes in men (37). This indicates that sex hormones play a complex and significant role in the development of diabetes. Additionally, increased fat mass in women, elevated levels of circulating non-esterified fatty acids, higher lipid content in muscle cells, and lower skeletal muscle mass are potential mechanisms underlying the differences in diabetes susceptibility between sexes. These factors contribute to women being less prone to insulin resistance compared to men. Although the incidence of diabetes appears to be higher in men, some risk factors seem to have a stronger association with diabetes in women than in men. For instance, hypertension, hyperlipidemia, and smoking are risk factors that may be more closely linked to diabetes in women (34, 35, 38–40). In this study, the association between MAP and IFG was relatively strong in women. Therefore, monitoring fluctuations in MAP among women may potentially yield better outcomes in the prevention and control of glucose metabolism abnormalities.

Compared to previously published studies, our research was a large-sample cohort study based on a Chinese population undergoing health examinations, with a follow-up period ranging from approximately 2 to 6 years. Our study provided robust evidence regarding the relationship between MAP and the risk of prediabetes, with a particular focus on addressing potential confounding factors and biases. However, there are limitations that should be considered. Firstly, as an observational study, it differs from randomized controlled trials, and thus, the results cannot establish a causal relationship between MAP and IFG. Therefore, careful interpretation of the findings is essential. Additionally, despite efforts to adjust for significant confounding factors, there may still be unmeasured confounders associated with the risk of prediabetes. However, for several reasons, the impact of these potential unmeasured confounders on the study results may be minimal (1): The results of subgroup analyses and sensitivity analyses remained consistent after adjusting for various types of covariates and different populations (2); The E-value was used to assess potential unmeasured confounding factors, and the results obtained were greater than the currently reported association strength related to prediabetes events. Furthermore, this study primarily included individuals from health examination populations in China, which may limit the generalizability of the findings and highlights the need for similar studies in different populations. Despite these limitations, in the future, we aim to confirm these findings through well-designed, multi-center prospective cohort studies.

## Conclusion

Our findings suggested a potential association between MAP levels and IFG status. The observed nonlinear relationship between MAP levels and the risk of IFG events indicated that controlling the rise in MAP levels may be more effective for the prevention of IFG when MAP is below 103.23 mmHg. Interaction analyses revealed that the association of MAP with IFG status was more pronounced in the younger adult group and among women. These findings underscore the importance of integrating demographic-specific considerations into clinical and public health strategies, enabling more precise risk monitoring and targeted interventions to mitigate IFG and prevent diabetes progression.

## Data Availability

The datasets presented in this study can be found in online repositories. The names of the repository/repositories and accession number(s) can be found below: The original data can be accessed through the Dryad data repository at https://datadryad.org/stash/dataset/doi:10.5061/dryad.ft8750v.
