# Correction: Elevated mean arterial pressure and risk of impaired fasting glucose: a multicenter cohort study revealing age and sex interactions

**DOI:** 10.3389/fendo.2025.1649984

**Published:** 2025-07-21

**Authors:** Yuye Lin, Junzhong Zou, Miaoling Hong, Xudong Huang, Juan Wu

**Affiliations:** ^1^ Department of Clinical Laboratory, Jieyang People’s Hospital, Jieyang, Guangdong, China; ^2^ Precision Medicine Centre, Puning People’s Hospital, Puning, Guangdong, China

**Keywords:** mean arterial pressure, impaired fasting glucose, cohort study, age interaction, sex interaction

There was a mistake in **Table 3** as published. The range “≥103.23–” should be revised to “≥103.23” for accuracy. The corrected **Table 3** appears below.

**Table 3:** Threshold effect of MAP on the incidence of IFG.

MAP (mmHg)	HR (95%CI)	*P* value
<103.23	1.014 (1.012–1.016)	< 0.001
≥103.23	1.008 (1.004–1.013)	< 0.001
Likelihood Ratio test		<0.001

During the final proofreading stage, we identified a non-citable reference in our manuscript. While we previously removed the relevant content from the background section, we recognize that the 2nd sentences in Paragraph 2 of the discussion section also requires deletion.

A correction has been made to the 2nd and 3rd sentences in Paragraph 2 of the **Discussion** section:

“For instance, a cohort study of a Chinese population undergoing physical examinations has confirmed the positive association between MAP and diabetes (14)”.

The original version of this article has been updated.

