# Supplementary material for: Elevated mean arterial pressure and risk of impaired fasting glucose: a multicenter cohort study revealing age and sex interactions
Source: Front Endocrinol (Lausanne). 2025 Jun 18;16:1580036. doi: 10.3389/fendo.2025.1580036 (PMC12213370; doi:10.3389/fendo.2025.1580036)
Supplement: Supplementary file 1 [file Table1.docx]

**Table S1 The association between MAP and the risk of IFG among different population**

| **Population** | **Model 1** | | **Model 2** | | **Model 3** | |
| --- | --- | --- | --- | --- | --- | --- |
|  | **HR (95% CI)** | ***P* value** | **HR (95% CI)** | ***P* value** | **HR (95% CI)** | ***P* value** |
| MAP (Per 10-mmHg increase) |  |  |  |  |  |  |
| Sensitivity 1 | 1.38 (1.35–1.42) | <0.001 | 1.13 (1.10–1.17) | <0.001 | 1.13 (1.10–1.16) | <0.001 |
| Sensitivity 2 | 1.37 (1.34–1.41) | <0.001 | 1.14 (1.11–1.17) | <0.001 | 1.13 (1.10–1.16) | <0.001 |
| Sensitivity 3 | 1.38 (1.37–1.40) | <0.001 | 1.14 (1.13–1.16) | <0.001 | 1.15 (1.13–1.18) | <0.001 |

Note: BMI, body mass index; SBP, systolic blood pressure; DBP, diastolic blood pressure; FPG, fasting plasma glucose; TC, total cholesterol; HDL-C, high-density lipoprotein cholesterol; LDL-C, low-density lipoprotein cholesterol; ALT, alanine aminotransferase; AST, aspartate aminotransferase; BUN, blood urea nitrogen; CCr, creatinine; TG, triglyceride; MAP, mean arterial pressure; IFG, impaired fasting glucose; 95% CI, 95% confidence interval; HR, hazard ratio

Sensitivity 1: Results for nonsmokers

Sensitivity 2: Results for nondrinkers

Sensitivity 3: Results based on original data

Model 1: Not adjusted for any confounders.

Model 2: Adjusted for age, sex, BMI, FPG.

Model 3: Adjusted for Model 2+TC, TG, LDL, HDL, ALT, AST, BUN, CCR, family history of diabetes, smoking status (not for the nonsmokers) and drinking status (not for the nondrinkers).
